# GRADE in Systematic Reviews of Acupuncture for Stroke Rehabilitation: Recommendations based on High-Quality Evidence

**DOI:** 10.1038/srep16582

**Published:** 2015-11-12

**Authors:** Zhang Xin, Liu Xue-Ting, Kang De-Ying

**Affiliations:** 1 Department of Integrated Traditional Chinese and Western Medicine, West China Hospital, Sichuan University, Chengdu 610041, P.R. China; 2 Department of Evidence-Based Medicine and Clinical Epidemiology, West China Hospital, Sichuan University, Chengdu 610041, P.R. China

## Abstract

Systematic reviews (SRs) of randomized controlled trials (RCTs) have demonstrated acupuncture’s effectiveness in stroke rehabilitation. The current study reviews the quality of evidence in SRs of acupuncture in stroke rehabilitation, and rates the strength of recommendation for its use based on this evidence using the GRADE (grading of recommendations, assessment, development and evaluations) approach. A comprehensive literature search was performed using multiple databases (e.g., Medline, Embase) with advanced search strategies. Two authors independently selected articles, collected data, and assessed the methodological quality of each identified SR according to AMSTAR (a measurement tool to assess systematic reviews) and OQAQ (Oxman and Guyatt’s overview quality assessment questionnaire). Outcomes related to stroke rehabilitation were evaluated. SRs of high methodological quality (AMSTAR score ≥9 and OQAQ score ≥7) were graded using GRADE. Ultimately, acupuncture yields benefits in stroke rehabilitation (neurological function improvement: RR = 1.34; swallowing improvement: RR = 1.61, 1.49, 1.07; disability: SMD = 0.49 or 0.07). Poor evidentiary quality and insufficient information about harm led to weak recommendations. In conclusion, acupuncture may improve stroke rehabilitation, as the GRADE approach indicated a weak recommendation for acupuncture’s usage in this context.

Stroke has increased the economic burden imposed on health care systems, accounting for 2% to 4% of total health care costs^1^, and is a major cause of death and permanent disability worldwide^2^. More than half of the patients who suffer from stroke become moderately to severely disabled^3^,^4^. Stroke is also the leading cause of death and long-term disability in China^5^,^6^, where the rising incidence of stroke has created a serious public health problem^7^,^8^. Post-stroke patients present with musculoskeletal, sensorimotor, perceptual, and cognitive deficits. Interventions intended to reduce pain and spasticity, as well as to increase range of motion (ROM), muscle force, mobility, ambulation, functionality, physical fitness, and quality of life, can be used for stroke rehabilitation^9^. Acupuncture, a component of traditional Chinese medicine, is an accepted complementary treatment in stroke rehabilitation both in Asian countries^10^,^11^ and the West^12^,^13^ due to its effects on spasticity, loss of function, loss of mobility, depression, aphasia, hemiplegia and pain reduction^14^. Therefore, the use of acupuncture has been advocated by reviews^14^ and Stroke Engine^14^ and has been included in guidelines^9^ pertaining to stroke rehabilitation.

Systematic reviews (SRs) of high-quality randomized controlled trials (RCTs) are considered the best evidence regarding specific healthcare interventions^15^,^16^. SRs, particularly high-quality reviews, provide clinicians with more reliable findings and enable conclusions to be drawn and decisions to be made regarding both patient care and health policy^15^,^17^,^18^. Many SRs of RCTs regarding acupuncture have been published with conflicting results, as some studies have described significant beneficial effects^9^,^14^, whereas others have failed to demonstrate any effects in the setting of stroke rehabilitation^10^,^14^,^19^,^20^,^21^,^22^,^23^. The Grading of Recommendations Assessment, Development and Evaluation (GRADE) approach^24^ is designed to rate the quality of a body of evidence and can be applied to evaluate SRs and other forms of evidence, including health technology assessments, and to determine the strengths of any relevant guidelines or recommendations^24^,^25^,^26^,^27^. The GRADE system clearly distinguishes between evidence quality and recommendation strength and takes into account other factors in addition to evidence to suggest appropriate therapeutic approaches^25^, resulting in a recommendation for or against an intervention that is based on whether the potential benefits of said intervention outweigh the potential harm caused or burden imposed by the intervention^24^, as well as on patient values and preferences.

Despite the availability of numerous publications regarding the use of acupuncture in stroke rehabilitation^10^,^14^,^19^,^20^,^21^,^22^,^23^, the evidence included in these SRs has not been evaluated systematically via the GRADE approach^28^,^29^. The aims of the current study were to review the quality of evidence in SRs of acupuncture in stroke rehabilitation, and to rate the strength of recommendation for its use based on this evidence using the GRADE approach.

## Methods

### Criteria for Inclusion

#### PICOS approach

The Population, Intervention, Comparator, Outcome and Study design (PICOS) approach was used to frame our research objectives.

### Study Design

SRs containing at least one RCT were included in this study.

### Study Participants

Patients with either hemorrhagic or ischemic stroke, at any stage or severity (including patients with cerebral infarction, intracerebral hemorrhage, cerebral embolism, or unclassified stroke), who were either (1) diagnosed via brain computed tomography (CT) scan or brain magnetic resonance imaging (MRI) or (2) diagnosed clinically according to the World Health Organization definition (rapidly developing focal or global disturbances of cerebral function lasting more than 24 hours or resulting in death, with no other apparent cause of vascular origin^23^), regardless of age, sex or neurological deficit severity.

### Intervention

Either traditional acupuncture, which entails the insertion of needles at classical meridian points, or contemporary acupuncture, which entails the insertion of needles at non-meridian points or trigger points, was utilized, regardless of the source of stimulation (hand stimulation, ear acupuncture, abdominal acupuncture, wrist-ankle needle, scalp acupuncture, fire needle, moxibustion with a warming needle, or electrical stimulation). SRs in which the acupuncture treatment did not involve needling, such as those using point injection, acupressure, laser acupuncture, tap-pricking or cupping on pricked superficial blood vessels, were excluded.

### Comparison

The control interventions included sham acupuncture, placebo acupuncture or other conventional treatments (including Western medical treatments, traditional Chinese medical treatments except acupuncture, physical therapy, occupational therapy, and speech therapy). Sham treatment included the following: (1) needle pricking on the skin surface (needles placed close to but not at the acupuncture points)^30^ or (2) subliminal skin electro-stimulation via electrodes attached to the skin. Placebo acupuncture referred to a needle being attached to but not penetrating the skin (the needle was applied to the same acupuncture points as in the treatment)^30^. SRs in which acupuncture treatment on an affected side was compared with that on an unaffected side were also considered for inclusion.

### Outcome Measures

We convened a panel of twelve experts in neurology, neurosurgery, cerebrovascular disease, acupuncture and traditional Chinese medicine at our hospital. These experts collected as many outcomes associated with stroke rehabilitation as possible and subsequently rated each outcome numerically from 1 to 9 points based on clinical importance (1: of least importance; 9: of most importance) and privately recorded their judgments. Generally, outcomes of long-term patient interest, as well as acupuncture-associated outcomes (bent needle, stuck needle, broken needle, fainting, injury to important organs, infection, bleeding), were defined as important outcomes. The individual judgments were then aggregated statistically to derive the median score for each outcome. We subsequently classified the importance of each outcome. Three outcome categories were specified based on their importance regarding clinical decision making: critical (median score of 7 to 9), important but not critical (median score of 4 to 6) and limited importance (median score of 1 to 3)^24^ (Table 1). Critical and important outcomes were used for decision-making and were included in the evidence profile^24^.

### Literature Sources and Search Strategy

We searched “pre-appraised” evidence resources (defined as resources that underwent a filtering process to include only high-quality studies; they are regularly updated so the evidence available via these resources is current^31^) to collect SRs from their inception until September 2014; the websites used are listed in the supplemental methods.

An extensive literature search was conducted using the following databases: Medline (Ovid, 1966-2014.9), Embase (Ovid, 1974-2014.9), the Cochrane Database of Systematic Reviews (Ovid, 1991-2014.9), Psych-info (-2014.9), ScienceDirect (-2014.9), the China National Knowledge Infrastructure/China Academic Journals Full-text Database (CNKI, 1994-2014.9), the Chinese Biomedical Literature Database (CBM, 1978-2014.9), the Chinese Scientific Journals Database (VIP, 1989-2014.9), the Traditional Chinese Medicine (TCM) Database (1949-2014.9) and the Wanfang Database (1998-2014.9). No language restriction was imposed during the study search. The search strategies used in Medline (Ovid) are listed in Appendix 1 and were appropriately adapted for the other databases and the Chinese electronic databases using Chinese terms. We manually searched through four Chinese journals relevant to acupuncture (Chinese Acupuncture and Moxibustion, the Journal of Clinical Acupuncture and Moxibustion, Acupuncture Research and the Shanghai Journal of Acupuncture and Moxibustion) for articles published between 1980 and November 2014. We checked the reference lists of all relevant SRs identified, and their authors were contacted as a means of identifying additional relevant SRs.

### SR Selection

Two authors (Zhang X and Liu XT) independently checked the titles and abstracts of the articles and retrieved the full texts of potentially eligible articles for further review based on the selection criteria outlined above. In cases of disagreement between the two authors, a third member of our research group (Kang DY) reviewed the information to determine whether it should be included or excluded.

### Selecting SRs of High Methodological Quality

The methodological quality of each SR was independently assessed by two reviewers using “A measurement tool to assess the methodological quality of systematic reviews” (AMSTAR^32^) and the Oxman-Guyatt Overview Quality Assessment Questionnaire (OQAQ)^33^, which contain 11 and 9 assessment criteria, respectively. Quality scores for both AMSTAR and OQAQ were calculated in accordance with the principles used in previous studies^34^,^35^,^36^ as follows: one point was awarded for each answer of “Yes”, and 0 points were awarded for all other cases. Total scores were obtained after the two tools were used. Additionally, Preferred Reporting Items for Systematic Reviews and Meta-Analyses (PRISMA)^37^ was utilized to assess report quality. SRs of high methodological quality were screened in accordance with the rating system of the “Canadian Agency for Drugs and Technologies in Health” (CADTH)^38^: we rated each SR as being of “high” (range 9–11), “moderate” (range 5–8), or “low” (range 0–4) quality based on the AMSTAR overall score. For the OQAQ, we rated studies with an overall score of ≥7 as high quality. All relevant articles were assessed by two authors (Zhang X and Liu XT) using the above two scales, and the SRs of high methodological quality (based on either AMSTAR or OQAQ) were used for data collection. Frequent, ongoing discussions among all the authors regarding any queries occurred throughout the rating process. Agreement validation and reliability were described in our previous article^39^.

### Data Collection

Using a standardized form, two authors (Zhang X and Liu XT) independently extracted data from the SRs, including participant characteristics (age, sex), intervention details, measured outcomes, number of included trials, sample sizes of each group, diagnostic criteria, TCM syndrome classification, study methodology, original RCT quality, and disease duration and state. The intervention details included meridian points, stimulation sources, drugs, medication doses, therapeutic regimens and treatment durations. Additional data and methodological information were obtained from the original RCT reports. We sought clarification from SR and RCT authors if an SR did not include clear descriptions of either the information or the methodologies used. Disagreements were resolved via discussion and consensus with a third researcher (Kang DY).

### The GRADE Approach

#### SR Evidence Quality

The GRADE approach^24^ was used to assess the quality of each available SR and the strength of a recommendation regarding the use of acupuncture in the setting of stroke. The results pertaining to the primary outcomes of the SRs that were initially considered high-quality evidence (an AMSTAR score ≥9 or an OQAQ score ≥7) were extracted, appraised critically and used to construct a body of evidence.

Two authors (Zhang X, a clinician with expertise in TCM, and Kang DY, a methodologist with expertise in both research methods and SRs) were trained to use the GRADE tool, which was obtained from the 22^nd^ Cochrane Colloquium (Hyderabad, India, from September 21^st^ to 26^th^, 2014). For each SR, the authors independently utilized the GRADE tool to evaluate the evidence pertaining to key outcomes. The methodological criteria by which evidence was upgraded or downgraded were dependent on five primary domains (risk of bias, inconsistency, indirectness, precision and publication bias), as well as the overall quality of the evidence (high, moderate, low, or very low)^24^.

### From SR Evidence to Recommendations

The second component of the GRADE approach entails determining the strength of a recommendation, i.e., the level of confidence that the desirable effects (benefits) of acupuncture outweighed the undesirable effects (harms) or vice versa^24^. The recommendations were assigned to one of two categories: strong recommendations and weak recommendations (Supplemental Table 1).

Evidence quality was considered along with three additional key factors—the best estimates of the magnitudes of the effects on both desirable and undesirable outcomes, the importance of the outcomes, and the confidence in the magnitudes of the estimates of the effects of acupuncture on the important outcomes (the overall quality of the evidence for the outcomes)^24^—that were collectively used to determine the strength of each recommendation.

### Statistical Analysis

The data were entered into EpiData 3.1^40^ and exported to SPSS-21.0 (SPSS, Inc., Chicago, IL, USA) for analysis. GRADEpro^41^ was used to grade both the quality of the evidence and the strength of the recommendations. Descriptive statistics, such as rate and proportion, were used for dichotomous data, and either means (standard deviations) or medians (ranges) were used for continuous data. We calculated the inter-rater reliability between the two reviewers for each GRADE domain and the overall quality of evidence using Kappa coefficients if the number of included SRs was sufficient. Two-tailed P values of 0.05 or lower were considered statistically significant.

## Results

### SR Selection

**Figure 1:**
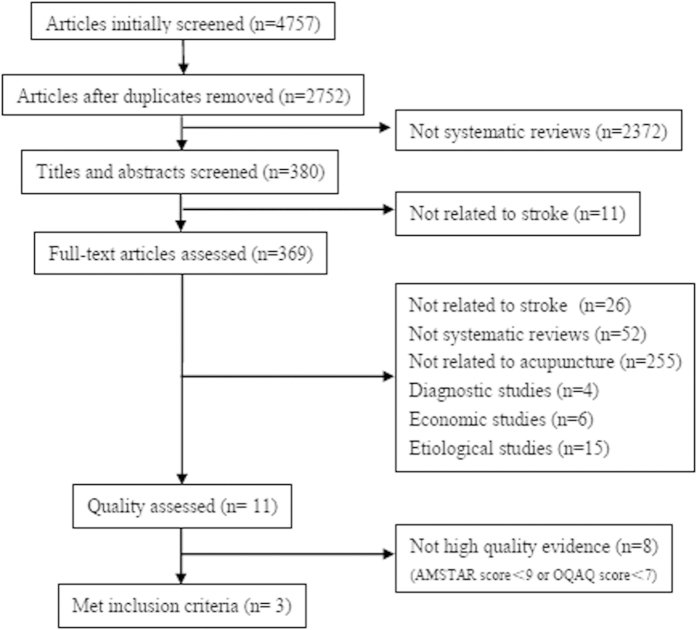
Flowchart: study selection.

A total of 4750 SRs were initially screened. Eleven SRs were finally selected for quality assessment using PRISMA, AMSTAR and OQAQ. Three SRs with high-quality evidence (an AMSTAR score ≥9 or an OQAQ score ≥7) that met the inclusion criteria were identified (Table 2). The detailed literature search process and study exclusion criteria are included in Fig. 1.

### Characteristics of the Included SRs and the Original RCTs

The three included SRs assessed 19 RCTs. The following interventions were investigated within the original RCTs and varied in duration, frequency and intensity:

- (1) Acupuncture (classical, electrical and sham)
- (2) Rehabilitation therapy
- (3) Physical therapy
- (4) Occupational therapy
- (5) Speech therapy
- (6) Traditional Chinese medicine
- (7) Use of aspirin

The outcome measures, intervention effects, risk of bias assessments and other characteristics of the included RCTs and SRs are presented in Tables 3 and 4.

### Quality Assessment of the Overall Body of SR Evidence using the GRADE Approach

Only quantitative analyses from the three included SRs (Supplemental Table 2) were used to determine the overall quality of the evidence supporting each specific recommendation via the GRADE approach. The evidence pertaining to the nine critical outcomes was downgraded to either low or moderate quality due to various limitations.

### Study Limitations

Although the included SRs were of good quality, indicated by a median AMSTAR score of 9 and a median OQAQ score of 8, the original RCTs were of poor quality. Four of the original RCTs^42^,^43^,^44^,^45^ that used neurological function as a primary outcome failed to report sufficient information to enable conclusions regarding whether the random sequence generation, allocation concealment, blinding or outcome data were adequate. The risk of bias assessments of the 19 included RCTs are included in Supplemental Table 2. The evidence comparing acupuncture plus conventional stroke rehabilitation (CSR) with CSR alone was also downgraded due to blinding (Table 3). Inadequate reporting increases the possibility of bias and decreases the validity of the GRADE approach.

### Inconsistencies in the Results

Regarding the global neurological deficit outcome for acupuncture plus conventional care vs. conventional care alone, inconsistencies were noted among the 4 studies^42^,^43^,^44^,^45^ in the results pertaining to different control interventions and combined interventions; there were also inconsistencies among the RCTs regarding the differences reported between the results for patients with ischemic stroke and those for patients with ischemic/hemorrhagic stroke. Statistical inconsistencies were observed among the 4 studies in the meta-analysis results, with an I^2^ = 63% (P = 0.04). As a result, we considered the level of inconsistency to be serious. Inconsistencies regarding the other outcomes were primarily attributed to differences in interventions, acupuncture details, stroke stages and methodological quality among the studies (downgraded by one level) (Table 3).

### Indirectness of the Evidence

The SR^46^ reporting global neurological deficit as a critical outcome included only patients with a stroke duration ≥1 month; consequently, the results from this study may only be applicable to patients recovering from stroke (Supplemental Table 2); however, we determined that the indirectness was not serious. Similarly, acupuncture helped improve swallowing during the sub-acute stage (stroke onset within 2 to 28 days) and improved both motor recovery and disability among patients who suffered either moderate or severe stroke (Supplemental Table 2).

### Precision

Because the total number of participants was small (<400)^24^, the 95% CI overlapped with no effect (i.e., an OR or RR of 1.0) and failed to exclude important benefits (an OR or RR increase of 25% or more)^24^, we subsequently downgraded the quality of the evidence of the six outcomes by one level based on imprecision (Table 3).

### Publication Bias

Publication bias could not be ruled out because of the limited number of trials pertaining to the included outcomes (Table 3).

### Recommendations

The quality of SR evidence was assessed using five components to grade the recommendations while taking into account the patients’ characteristics^24^ (Table 4). The small relative effects (RR < 2)^24^ of acupuncture on both desirable and undesirable outcomes were more likely to warrant a weak recommendation. Furthermore, the low overall quality of evidence regarding critical outcomes and the low confidence in the effects on other outcomes were considered critical (often causing long-term harm) and resulted in weak recommendations. The strength of the recommendations regarding the use of acupuncture in patients suffering from stroke is described in detail in Table 4.

## Discussion

Three high-quality SRs were identified, in which seventeen RCTs reporting nine critical outcomes with quantitative analyses were used to determine the overall quality of the evidence supporting the GRADE recommendation that acupuncture yields benefits in stroke rehabilitation (neurological function improvement, swallowing improvement and disability). Virtually none of the authors of the included SRs were aware of negative effects caused by acupuncture, which can occur even when it is performed by a well-trained, licensed acupuncturist. The weak strength of the recommendation to use acupuncture in stroke rehabilitation implies that the decision to prescribe acupuncture for a patient suffering from stroke symptoms and sequelae should be approached with caution. In this study, we did not rate the recommendations of the domains of values and preferences or resource use, as reliable data were unavailable.

Rating an overall body of evidence using the GRADE approach is becoming an important and recommended step in evidence synthesis initiatives^24^ and may improve the transparency of shared decision-making processes, particularly under conditions in which the quality of evidence is either low or unclear. When evaluating the available evidence, to perform a final quality assessment, the GRADE approach includes detailed scrutiny of the potential limitations within a whole body of evidence, considering factors such as risk of bias, result inconsistency, indirectness and imprecision (Table 3). Rating the quality of a body of evidence is valuable for end users (patients, clinicians, and policy makers) of evidence syntheses, as it serves as an indicator of the confidence they should have in the results. Additionally, producing this rating is a key step in translating a particular body of evidence into clinical practice^24^. Moreover, GRADE clearly differentiates evidence quality from recommendation strength. Recommendation evaluation occurs during the second step of the decision-making process, during which evidence quality is considered in light of other factors to enable both a correct and transparent judgment of recommendation strength. This approach is realistic because in a clinical setting, decisions regarding therapy cannot be made solely on the basis of evidence quality. To the best of our knowledge, this is the first study to apply the GRADE approach to evaluate SRs regarding the use of acupuncture in stroke rehabilitation.

Several organizations have summarized the evidence regarding the use of acupuncture in stroke rehabilitation; however, conflicting conclusions have been drawn. The Ottawa Panel^9^ developed guidelines, published in 2006, regarding the use of acupuncture in the management of adult patients suffering from stroke. The guidelines recommend acupuncture as an adjunct treatment to improve specific outcomes during both the acute and subacute stages of stroke rehabilitation. However, based on the results of several SRs, Stroke Engine^14^ concluded that acupuncture is not an effective treatment in the setting of stroke rehabilitation, as there is insufficient evidence to draw such a conclusion. Furthermore, the Evidence-based Review of Stroke Rehabilitation (EBRSR)^47^ summarized several SRs published over the past 15 years with conflicting results: some SRs determined that acupuncture provides beneficial effects in the rehabilitation of stroke patients, whereas others did not. In the EBRSR review, neither the methodological quality of each SR nor the quality of the evidence for each outcome across all SRs (i.e., the body of evidence for an outcome) were rated, and conflicting results were not explained; thus, this review may confuse clinicians who must make decisions regarding patients. In general, to help clinicians make evidence-based medical decisions, review authors should rate both the quality of available evidence and include informed recommendations. These recommendations entail balancing the desirable and undesirable consequences of a given treatment and may help both clinicians and policy makers make better decisions regarding patient care. Future research should therefore focus more on developing a system with which to synthesize SR evidence into a greater body of evidence as opposed to providing a brief summary of results.

We encountered several challenges using GRADE. During the first round of risk bias assessment, we struggled in determining how to integrate the quality assessments of the original RCTs into a whole body of evidence. Additionally, poor reporting on “sequence generation”, “incomplete outcome data”, “allocation concealment” and “selective reporting” downgraded the risk of bias^15^; in instances such as these, the overall body of evidence should be interpreted cautiously. Although the GRADE Handbook suggests that, in principle, evaluating the extent to which each RCT contributes toward estimating the magnitude of an effect (usually reflecting both study sample size and number of outcome events) is warranted^24^, when assessing our overall body of evidence with respect to discrepancies in the risk of bias assessments among the RCTs, the utilization of said contributions was both challenging and impractical. Moreover, shifting away from overall quality scores or summaries toward a component approach for the individual studies^15^ was found to be incongruous with forcing an overall assessment of the risk of bias for a group of RCTs. Additionally, our study identified more than 10 outcomes within the 19 included RCTs, which made it difficult to compare inconsistencies without a meta-analysis, as there was no I^2^ statistic with which to determine heterogeneity. Although a qualitative approach may be utilized to assess consistency across multiple studies (considering whether estimates were similar in terms of the magnitude and direction of an effect as well as its statistical significance), such an approach is both subjective and unreliable. Similarly, imprecision assessments across studies are particularly problematic in the absence of a meta-analysis. We included only outcomes with quantitative analyses. In addition to confidence intervals (CIs) and the lines of no effect, another criterion, the optimum information size (OIS), should be used to ensure adequate precision. OIS is defined by the number of patients generated by a conventional sample size calculation for a single trial. If the total number of patients included in an SR is less than the OIS, the quality of the SR should be downgraded due to imprecision^24^. The greater the difference between the number of subjects included in a potentially adequate RCT and the number of subjects included in an SR, the greater the probability of downgrading the SR due to imprecision. In this study, because of insufficient reporting regarding sample size calculation and a lack of data regarding Δ in the original RCTs^24^, the minimum OIS number was not obtained. The small sample sizes (n < 400) of the three critical outcomes may therefore support the decision to downgrade the quality of the evidence by one level^24^.

There are several limitations to this study. A highly sensitive search strategy was utilized to identify current SRs; however, only three SRs were included for grading. This low number may have been due to methodological quality. The purpose of utilizing GRADE was to obtain both valid and reliable information to guide evidence-based decisions. The production of both comprehensive and accessible pre-appraised resources supports the use of an evidence-based approach to decision making. If SRs are to be useful, serious consideration must be given to how they are conducted. When the SR evidence gathered is strong and its implications are clear, the generated body of evidence should subsequently influence decision-making and shape health policy. The ultimate test of an SR is whether conducting it produced confidence that it is evidence-based and that it accurately reflects a process during its various stages. Evaluating the validity of SRs should influence the analysis, interpretation and conclusions of the GRADE approach. In particular, an SR of invalid studies may produce a misleading GRADE result, yielding a narrow confidence interval (good precision) around the wrong intervention effect estimate. Therefore, only high-quality SRs were selected for our study. Furthermore, our results may have been biased, as our selection process missed primary articles included in lower-quality SRs (which were excluded during the selection process). The objective of this research was to synthesize evidence from SRs; therefore, we did not consider these potentially missing primary articles. Future research may synthesize the evidence from primary articles, such as RCTs and cohort studies, using the GRADE approach; the results of these two research studies could then be compared. Moreover, almost none of the authors of the included SRs were aware of the negative effects caused by acupuncture—even when it is performed by a well-trained, licensed acupuncturist—other than occasional bruising. Finally, we did not perform sensitivity analyses to explore the differences among the results.

Additionally, although two reviewers independently utilized the GRADE tool to rate the SRs, an agreement assessment was not performed because of the small number of SRs included in this analysis. It is important to emphasize that some subjectivity exists when assessing both the quality of evidence and the strength of a recommendation. However, our decision process was transparent, and frequent discussions took place among all authors regarding any queries. Finally, we did not assess publication bias, as we did not have a sufficient number of studies with which to formally evaluate it. Thus, publication bias may have existed in our findings, although a sensitive search strategy was utilized.

## Conclusion

In summary, we systematically assessed current evidence from SRs regarding the use of acupuncture in patients suffering from stroke. Moreover, an innovative approach was utilized to assess the quality of the SR evidence and the strength of recommendations pertaining to specific clinical procedures. Recent SRs that have evaluated RCTs describe the potential benefits of using acupuncture in stroke patients to improve rehabilitation; however, the overall body of evidence was found to be of low quality. Our critical appraisal of the evidence using the GRADE approach resulted in the formulation of a weak recommendation regarding the use of acupuncture in the setting of stroke rehabilitation. High-quality, well-designed SRs and RCTs are warranted to support the utilization of acupuncture in the setting of stroke rehabilitation.

## Additional Information

**How to cite this article**: Xin, Z. *et al.* GRADE in Systematic Reviews of Acupuncture for Stroke Rehabilitation: Recommendations based on High-Quality Evidence. *Sci. Rep.* **5**, 16582; doi: 10.1038/srep16582 (2015).

## Supplementary Material

Supplementary Information

## Figures and Tables

**Table 1:** Rating scale for outcome ranking according to clinical importance.

Importance	Measure
Critical*	Death (Mortality rate; Fatality rate; DALY; Death rate)
	Disabilities
	Physiological function (Language function; Sensory function; Swallowing function; Motor function; Strength of respiratory muscles)
	Stroke-associated outcomes (Stroke recurrence; Intracranial/Extracranial hemorrhage; Modified Rankin Scale^48^; Barthel Index^48^; Modified Ashworth Scale (MAS)^49^; Scandinavian Stroke Scale (SSS)^50^; National Institutes of Health Stroke Scale (NIHSS)^51^)
	Quality of life (QOL)
Important†	Acupuncture-associated outcomes (Bent needle; Stuck needle; Broken needle; Fainting; Injury to important organs; Infection; Bleeding)
	Withdrawal
Not important‡	Anxiety and depression
	Cognition of disease
	Patient satisfaction

^*^Critical for making a decision and included in the evidence profile.

^†^Important for making a decision and included in the evidence profile.

^‡^Not important for making a decision and not included in the evidence profile.

**Table 2:** SR evidence included in the GRADE approach.

SR	PRISMAscore	AMSTAR score	OQAQscore
Wu 2006^46^	24	9	9
Xie 2008^53^	25	9	8
Sze 2002^54^	25	9	8
SR, systematic review			

**Table 3:** Quality assessment and summary of findings using the GRADE approach.

Outcomes	Patients(n)	IncludedRCTs (n)	FU	Quality assessment						Summary of findings		
				Risk of bias	Inconsistency	Indirectness	Imprecision	Publication bias	Overall quality of evidence	Study event rates(%)		Relative effect [95% CI]
										Control	Acupuncture	
Acupuncture+CC vs. CC												
*Neurological function(CSRS)*	352	4 RCT^42^,^43^,^44^,^45^	30-90 d	No serious risk of bias^1^	Serious^2^	No serious indirectness	Serious^4^	Undetected^5^	⊕ΘΘΘLOW^1^^,^^2^^,^^3^^,^^4^^,^^5^	118/169 (69.8%)	173/183 (94.5%)	6.55*[1.89—22.76)]^6^
*Swallowing function* (recovery rate)	66	1 RCT^56^	na	Serious risk of bias^8^	No serious inconsistency	No serious indirectness	Serious^4^	Undetected^5^	⊕ΘΘΘLOW^4^^,^^5^	7/32 (21.9%)	12/34 (35.3%)	1.61†[0.73—3.58]^7^
*Swallowing function* (markedly improved rate)	66	1 RCT^56^	na	Serious risk of bias^8^	No serious inconsistency	No serious indirectness	Serious^4^	Undetected^5^	⊕ΘΘΘLOW^4^^,^^5^	17/32 (53.1%)	27/34 (79.4%)	1.49†[1.03—2.16]^6^
*Swallowing function* (improved rate)	66	1 RCT^56^	na	Serious risk of bias^8^	No serious inconsistency	No serious indirectness	Serious^4^	Undetected^5^	⊕ΘΘΘLOW^4^^,^^5^	29/32 (90.6%)	33/34 (97.1%)	1.07†[0.94—1.21]^7^
Acupuncture+CSR vs. CSR												
*Motor recovery change*	481	6 RCTs^12^,^57^,^58^,^59^,^60^,^61^	7 d-1 yr	Serious risk of bias^10^	Serious^9^	No serious indirectness	No serious imprecision	Undetected^5^	⊕ΘΘΘLOW^5^^,^^9^^,^^10^	—	—	0.06††[1.12—0.24]
*Disability change*	481	6 RCTs^12^,^57^,^58^,^59^,^60^,^61^	7 d-1 yr	Serious risk of bias^10^	Serious^9^	No serious indirectness	No serious imprecision	Undetected^5^	⊕ΘΘΘLOW^5^^,^^9^^,^^10^	—	—	0.49††[0.03—0.96]
Acupuncture+CC vs. CC												
*Disability*	488	4 RCTs^62^,^64^,^65^,^66^	2 d-40 d	No serious risk of bias	Serious^9^	No serious indirectness	No serious imprecision	Undetected^5^	⊕⊕⊕ΘMODERATE^5^^,^^9^	157/223 (70.4%)	258/265 (97.4%)	12.5*[4.3—36.2]
Real Acupuncture+CSR vs. Sham Acupuncture+CSR												
*Disability change*	254	2 RCTs^58^,^67^	3 w-12 mo	No serious risk of bias	Serious^9^	No serious indirectness	Serious^4^	Undetected^5^	⊕ΘΘΘLOW^4^^,^^5^^,^^9^	—	—	0.07††[-0.34—0.48]
*Motor recovery change*	270	3 RCTs^55^,^58^,^67^	3 w-12 mo	No serious risk of bias	Serious^9^	No serious indirectness	Serious^4^	Undetected^5^	⊕ΘΘΘLOW^4^^,^^5^^,^^9^	—	—	-0.06††[-1.24—1.12]

CI, confidence interval; *OR, odds ratio; ^†^RR, relative risk; ^††^SMD, standardized mean difference; na, not available; CSRS, Chinese Stroke Recovery Scale; CSR, conventional stroke rehabilitation; CC, conventional care; FU, follow-up.

^1^The authors of the SR^46^ stated no trial described the method of randomization, allocation concealment wwas unclear in all included trials, and only one trial reported that participants were blinded but did not describe the method in detail. No information on blinding was available in the remaining three trials.

^2^Inconsistencies were found among the 4 studies in the meta-results with a substantially large I^2^ (I^2^ = 63%, P = 0.04).

^3^Differences between study populations (Ischemic/hemorrhagic) and durations of stroke.

^4^The sample size was less than 400, the 95% CI overlapped with no effect (i.e., an OR or RR of 1.0), and CI failed to exclude important benefits (an OR or RR increase of 25% or more).

^5^It was not possible to check publication bias because of the limited number of trials for this outcome.

^6^The 95% CI failed to exclude important benefits (an OR increase of 25% or more).

^7^The 95% CI overlapped with no effect (CI includes RR of 1.0), and the CI failed to exclude important benefits (an RR increase of 25% or more).

^8^Downgraded for blinding (blinding of participants and personal and outcome assessment).

^9^Inconsistency was mainly due to differences in intervention, details of acupuncture and methodological quality among these studies.

^10^Risk of bias was mainly due to blinding.

**Table 4:** From SR evidence to recommendations.

Treatmentstrategies	Outcomes	Patients characteristics			Factors determining recommendations							
		Stroke type	Severity onentry	Stage	Best estimates of the magnitude of effects			Importanceof outcomes	Quality of evidence	Quality ofevidence acrosscritical outcomes	Balance of benefitsand harms†	Strength ofrecommendation
					Point estimateof Relative effects	Large magnitudeof effect*	Directionof effect					
Acupuncture+CC	Neurological function	Ischemic/hemorrhagic	L, Md, S	Recovery	OR=6.55 (RR=1.34)	No	Favors acupuncture	Critical	Low	Low	Uncertain‡	Weak↑?
	Swallowing function	Ischemic/hemorrhagic	L, Md, S	Subacute	RR=1.61, 1.49, 1.07	No	Favors acupuncture	Critical	Low			
Acupuncture+CSR	Motor recovery	Ischemic/hemorrhagic	Md, S	Acute, subacute and recovery	SMD = 0.06, -0.06	Unclear†	Favors acupuncture or CSR	Critical	Low	Low	Uncertain‡	Weak↑?
	Disability	Ischemic/hemorrhagic	Md, S	Acute, subacute and recovery	SM = 0.49, 0.07	Unclear†	Favors acupuncture	Critical	Moderate and Low			
Acupuncture+CC	Disability	Ischemic/hemorrhagic	L,Md, S	Acute, subacute and recovery	OR = 12.5, RR = 1.37	Unclear†	Favors acupuncture	Critical	Moderate and Low	Low	Uncertain‡	Weak↑?

OR, odds ratio; RR, relative risk; CSR, conventional stroke rehabilitation; CC, conventional care; L, light; Md, moderate; S, severe.

^↑?^Symbolic representation of weak recommendation for an intervention.

^*^GRADE definition of magnitude of effect: large, RR > 2 or <0.5; very large, RR > 5 or <0.2. We converted OR to RR when assessing the magnitude of an OR effect.

^†^No current definition of a large effect for this continuous variable.

^‡^Insufficient information from SRs and original RCTs for assessing harm.

## References

[b1] Donnan G. A., Fisher M., Macleod M. & Davis S. M. Stroke. Lancet 371, 1612–1623 (2008). 18468545 10.1016/S0140-6736(08)60694-7

[b2] Murray C. J. & Lopez A. D. Mortality by cause for eight regions of the world: Global burden of disease study. Lancet 349, 1269–1276 (1997). 9142060 10.1016/S0140-6736(96)07493-4

[b3] Wade D. T. & Hewer R. L. Functional abilities after stroke: Measurement, natural history and prognosis. J Neurol Neurosur Ps 50, 77–182 (1987). 10.1136/jnnp.50.2.177 PMC1031489 3572432

[b4] Jorgensen H. S. *et al.* Outcome and time course of recovery in stroke. Part II: Time course of recovery. The Copenhagen Stroke Study. Arch Phys Med Rehab 76, 406–412 (1995). 10.1016/s0003-9993(95)80568-0 7741609

[b5] Wang Y. J. *et al.* Chinese guidelines for the secondary prevention of ischemic stroke and transient ischemic attack 2010. Cns Neurosci Ther 18, 93–101 (2012). 22313945 10.1111/j.1755-5949.2011.00290.x PMC6493434

[b6] Liu L., Wang D., Wong K. S. & Wang Y. Stroke and stroke care in china: Huge burden, significant workload, and a national priority. Stroke 42, 3651–3654 (2011). 22052510 10.1161/STROKEAHA.111.635755

[b7] Liu M. *et al.* Stroke in China: Epidemiology, prevention, and management strategies. Lancet Neurol 6, 456–464 (2007). 17434100 10.1016/S1474-4422(07)70004-2

[b8] Zhao D. *et al.* Epidemiological transition of stroke in China: Twenty-one-year observational study from the Sino-Monica-Beijing project. Stroke 39, 1668–1674 (2008). 18309149 10.1161/STROKEAHA.107.502807

[b9] The Ottawa Panel. Ottawa Panel Evidence-Based Clinical Practice Guidelines for Post-Stroke Rehabilitation. Top Stroke Rehabil 13(2), 1–269 (2006). 10.1310/3TKX-7XEC-2DTG-XQKH 16939981

[b10] Wu P., Mills E., Moher D. & Seely D. Acupuncture in poststroke rehabilitation: A systematic review and meta-analysis of randomized trials. Stroke 41, e171–179 (2010). 20167912 10.1161/STROKEAHA.109.573576

[b11] Lee J. D. *et al.* The cerebrovascular response to traditional acupuncture after stroke. Neuroradiology 45, 780–784 (2003). 12942221 10.1007/s00234-003-1080-3

[b12] Hopwood V. & Lewith G. T. Does acupuncture help stroke patients become more independent? J Altern Complement Med 11(1), 175–177 (2005). 15750379 10.1089/acm.2005.11.175

[b13] Acupuncture Introduction. Stroke Engine. http://www.strokengine.ca/intervention/acupuncture/. Accessed August 27, 2015.

[b14] Acupuncture: Best Practices. *Stroke Engine*. www.strokengine.ca/best_practice/acupuncture-best-practices/. Accessed April 21, 2015.

[b15] Cochrane Handbook for Systematic Reviews of Interventions Version 5.1.0. *The Cochrane Collaboration*, 2011. http://www.cochrane-handbook.org.Accessed May 11, 2014.

[b16] The Evidence Pyramid. *The Medical Research Library of Brooklyn*. http://library.downstate.edu/EBM2/2100.htm. Accessed March 20, 2014.

[b17] Young D. Policymakers, experts review evidence-based medicine. Am J Health-Syst Ph 62, 342–343 (2005). 10.1093/ajhp/62.4.0342 15745880

[b18] Yang M. *et al.* A systematic review on natural medicines for the prevention and treatment of Alzheimer’s disease with meta-analyses of intervention effect of Ginkgo. Am J Chinese Med 42, 505–521 (2014). 10.1142/S0192415X14500335 24871648

[b19] Kim M-k., Choi T.-Y., Lee M. S., Lee H. & Han C.-h. Contralateral acupuncture versus ipsilateral acupuncture in the rehabilitation of post-stroke hemiplegic patients: A systematic review. Bmc Complem Altern M 10, 41 (2010). 10.1186/1472-6882-10-41 PMC2924268 20673364

[b20] Zhang S. H., Liu M., Asplund K. & Li L. Acupuncture for acute stroke. Cochrane Db Syst Rev, Cd003317 (2005). 10.1002/14651858.CD003317.pub2 15846657

[b21] Sze F. K., Wong E., Or K. K., Lau J. & Woo J. Does acupuncture improve motor recovery after stroke? A meta-analysis of randomized controlled trials. Stroke 33, 2604–2619 (2002). 12411650 10.1161/01.str.0000035908.74261.c9

[b22] Park J., Hopwood V., White A. R. & Ernst E. Effectiveness of acupuncture for stroke: A systematic review. J Neurol 248, 558–563 (2001). 11517996 10.1007/s004150170132

[b23] Hatano S. Experience from a multicenter stroke register: a preliminary report. B World Health Organ. 54, 541–53 (1976). PMC2366492 1088404

[b24] Schünemann H., Brożek J., Guyatt G. & Oxman A., editors. GRADE handbook for grading quality of evidence and strength of recommendations. The GRADE Working Group. http://www.guidelinedevelopment.org/handbook. Accessed March 20, 2014.

[b25] Guyatt G. H. *et al.* Grade: An emerging consensus on rating quality of evidence and strength of recommendations. BMJ 336, 924–926 (2008). 18436948 10.1136/bmj.39489.470347.AD PMC2335261

[b26] Guyatt G. H. *et al.* What is “quality of evidence” and why is it important to clinicians? BMJ 336, 995–998 (2008). 18456631 10.1136/bmj.39490.551019.BE PMC2364804

[b27] Guyatt. *et al.* Going from evidence to recommendations. BMJ 336, 1049–1051 (2008). 18467413 10.1136/bmj.39493.646875.AE PMC2376019

[b28] Kavanagh B. P. The grade system for rating clinical guidelines. Plos Med. 6, e1000094 (2009). 19753107 10.1371/journal.pmed.1000094 PMC2735782

[b29] Atkins D. *et al.* Systems for grading the quality of evidence and the strength of recommendations ii: Pilot study of a new system. Bmc Health Serv Res 5, 25 (2005). 15788089 10.1186/1472-6963-5-25 PMC1084246

[b30] Furlan A. D. *et al.* Acupuncture and dry-needling for low back pain. Cochrane Db Syst Rev, Cd001351 (2005). 10.1002/14651858.CD001351.pub2 PMC12145953 15674876

[b31] Dicenso A., Bayley L. & Haynes R. B. Accessing pre-appraised evidence: Fine-tuning the 5s model into a 6s model. Evidence Based Nurs 12, 99–101 (2009). 10.1136/ebn.12.4.99-b 19779069

[b32] Shea B. J. *et al.* Development of AMSTAR: A measurement tool to assess the methodological quality of systematic reviews. Bmc Med Res Methodol 7, 10 (2007). 17302989 10.1186/1471-2288-7-10 PMC1810543

[b33] Oxman A. D. & Guyatt G. H. Validation of an index of the quality of review articles. J Clin Epidemiol 44, 1271–1278 (1991). 1834807 10.1016/0895-4356(91)90160-b

[b34] Sequeira-Byron P., Fedorowicz Z., Jagannath V. A. & Sharif M. O. An AMSTAR assessment of the methodological quality of systematic reviews of oral healthcare interventions published in the journal of applied oral science (JAOS). J Appl Oral Sci 19, 440–44735 (2011). 21986647 10.1590/S1678-77572011000500002 PMC3984188

[b35] Shea B. J. *et al.* External validation of a measurement tool to assess systematic reviews (AMSTAR). Plos One 2, e1350 (2007). 18159233 10.1371/journal.pone.0001350 PMC2131785

[b36] Zhang X. *et al.* The external validity of randomized controlled trials of hypertension within china: From the perspective of sample representation. Plos One 8, e82324 (2013). 24324771 10.1371/journal.pone.0082324 PMC3855762

[b37] Liberati A. *et al.* The PRISMA statement for reporting systematic reviews and meta-analyses of studies that evaluate health care interventions: Ann Intern Med. 151, 65–94 (2009). 10.7326/0003-4819-151-4-200908180-00136 19622512

[b38] Canadian Agency for Drugs and Technologies in Health (CADTH). *Interventions Directed to Consumers*. http://www.cadth.ca/en/resources/rx-for change/interventions consumers. Accessed March 16, 2014.

[b39] Kang D. *et al.* Reliability and external validity of AMSTAR in assessing quality of TCM systematic reviews. Evid-Based Compl Alt 2012, e732195 (2012). 10.1155/2012/732195 PMC3292204 22454679

[b40] Lauritsen J. Epidata data entry, data management and basic statistical analysis system. Odense,Denmark: EpiData Association, 55–102 (2000).

[b41] Brożek J., Nowak A., Kunstman P. & Schünemann H. J. *GRADEpro Guideline Development Tool (G2DT*). http://www.guidelinedevelopment.org. Accessed March 12, 2014.

[b42] Dai C. Y. & Lu F. Efficacy observation on the treatment of sequelae of cerebral infarction in 46 patients by integrating Chinese with modern medicine. pract j int chinese mod med 10, 438 (1997).

[b43] Li X. Efficacy analysis of rehabilitation in the treatment of hemiplegia in 112 stroke patients. Chinese J Reh Theory P 3, 22–24 (1997).

[b44] Lun X., Peng Z. F. & Peng S. Z. Clinical observation on the treatment of sequelae of stroke by needling temporal three points. J Clin Acupunct Moxibust. 15, 8–9 (1999).

[b45] Wang Z. Y. Compared analyses among Chinese medicinal herbs, acupuncture and combination of them in the treatment of hemiplegia in stroke patients. Mod Reh. 5, 130 (2001).

[b46] Wu H. *et al.* Acupuncture for stroke rehabilitation. Cochrane Db Syst Rev 19, CD004131 (2006). 10.1002/14651858.CD004131.pub2 16856031

[b47] Norine F. *et al.* Miscellaneous Treatments. *Evidence-Based Review of Stroke Rehabilitation website*. http://www.ebrsr.com/sites/default/files/Chapter-20_Miscellaneous Treatments_FINAL_16ed.pdf. Accessed April 21, 2015.

[b48] Sulter G., Steen C. & De Keyser J. Use of the Barthel index and modified Rankin scale in acute stroke trials. Stroke 30, 1538–1541 (1999). 10436097 10.1161/01.str.30.8.1538

[b49] Bohannon R. W. & Smith M. B. Interrater reliability of a modified ashworth scale of muscle spasticity. Phys Ther 67, 206–207 (1987). 3809245 10.1093/ptj/67.2.206

[b50] Barber M., Fail M., Shields M., Stott D. J. & Langhorne P. Validity and reliability of estimating the scandinavian stroke scale score from medical records. Cerebrovasc Dis 17, 224–227 (2003). 14707426 10.1159/000075795

[b51] Goldstein L. B. & Samsa G. P. Reliability of the national institutes of health stroke scale extension to non-neurologists in the context of a clinical trial. Stroke 28, 307–310 (1997). 9040680 10.1161/01.str.28.2.307

[b52] Craig P. *et al.* Developing and evaluating complex interventions: The new medical research council guidance. BMJ 35, 337 (2008). 10.1136/bmj.a1655 PMC2769032 18824488

[b53] Xie Y., Wang L-p., He J-h. & Wu T. Acupuncture for dysphagia in acute stroke. Cochrane Db Syst Rev 16, CD006076 (2008). 10.1002/14651858.CD006076.pub2 PMC12147868 18646136

[b54] Sze FK-h., Wong E., Or K. K., Lau J. & Woo J. Does acupuncture improve motor recovery after stroke? A meta-analysis of randomized controlled trials. Stroke 33, 2604–2619 (2002). 12411650 10.1161/01.str.0000035908.74261.c9

[b55] Naeser M. A. *et al.* Real versus sham acupuncture in the treatment of paralysis in acute stroke patients: A ct scan lesion site study. Neurorehab Neural Re 6, 163–174 (1992).

[b56] Han J. C. An observation on the therapeutic effect of acupuncture for bulbar palsy after acute stroke. Henan J Pract Nerv Dis 7, 81–82 (2004).

[b57] Johansson K., Lindgren I., Widner H., Wiklund I. & Johansson B. Can sensory stimulation improve the functional outcome in stroke patients? Neurology 43, 2189–2189 (1993). 8232927 10.1212/wnl.43.11.2189

[b58] Gosman-Hedström G. *et al.* Effects of acupuncture treatment on daily life activities and quality of life a controlled, prospective, and randomized study of acute stroke patients. Stroke 29, 2100–2108 (1998). 9756589 10.1161/01.str.29.10.2100

[b59] Sällström S. *et al.* Acupuncture in the treatment of stroke patients in the subacute stage: A randomized, controlled study. Complement Ther Med 4, 193–197 (1996).

[b60] Wong A., Su T., Tang F., Cheng P. & Liaw M. Clinical trial of electrical acupuncture on hemiplegic stroke patients. Am J Phys Med Rehab 78, 117–122 (1998). 10.1097/00002060-199903000-00006 10088585

[b61] Sze, F. K. H., Wong E., Xiang Y. & Woo J. Does acupuncture have additional value to standard poststroke motor rehabilitation? Stroke 33(1), 186–194 (2002). 11779909 10.1161/hs0102.101815

[b62] Tang Q. S. & Sun S. T. Clinical, electrophysiological and biochemical changes following cranial acupuncture therapy to acute cerebral infarction. Beijing Trad Chin Med Univ Bull 19, 37–39 (1996).

[b63] Si Q. M., Wu G. C. & Cao X. D. Effects of electroacupuncture on acute cerebral infarction. Acupunct Electrother Res Int J 23, 117–124 (1998). 10.3727/036012998816356562 9789586

[b64] Jin Z. Q., Ku F. L., Chan S. X. & Chen G. X. Effect of acupuncture using acupoints of the du meridian on acute cerebral infarction. Acupunct Res 1, 5–7 (1999).

[b65] Li Q., Xiao G. Y. & Tung K. Y. Clinical study of the effect of cranial acupuncture on acute cerebral haemorrhage. J Chin West Med Integrat 19, 203–205 (1999). 11783265

[b66] Zhang Q. C., Lo L. B., Yu L., Chang L. T. & Chang Y. M. The effectiveness of acupuncture using “six acupoints for hemiplegia” method in the treatment of 145 acute stroke patients with middle-meridians abnormality. Clin Acupunct J 5, 46–48 (1999).

[b67] Johansson B. B. *et al.* Acupuncture and transcutaneous nerve stimulation in stroke rehabilitation a randomized, controlled trial. Stroke 32, 707–713 (2001). 11239191 10.1161/01.str.32.3.707

[b68] Chou TH Y. Y., Sun M. Y. & Yu C. K. 32 cases with ischemic stroke and haemorrhagic transformation treated with acupuncture. Acupunct Clin J 6, 6–7 (2000).

