# Supplementary material for: GRADE in Systematic Reviews of Acupuncture for Stroke Rehabilitation: Recommendations based on High-Quality Evidence
Source: Sci Rep. 2015 Nov 12;5:16582. doi: 10.1038/srep16582 (PMC4642304; doi:10.1038/srep16582)
Supplement: Supplementary Information [file srep16582-s1.doc]

**Recommendations for acupuncture in stroke patients**

**GRADE in Systematic Reviews of** **Acupuncture for Stroke** **Rehabilitation: Recommendations based on High-Quality Evidence**

Zhang Xin^1^,MD; Liu Xue-ting^2^,MD; Kang De-Ying^2*^,PhD

1. Department of Integrated Traditional Chinese and Western Medicine, West China Hospital, Sichuan University, Chengdu 610041, P. R. China.

2. Department of Evidence-Based Medicine and Clinical Epidemiology, West China Hospital, Sichuan University, Chengdu 610041, P. R. China.

**Supplemental Materials**

Supplemental methods. Search strategy

Supplemental table I. The strength of recommendations

Supplemental table II. Characteristics of included systematic reviews and their original RCTs

***Corresponding author**: Kang De-Ying.

E-mail address: [deyingkang@126.com](mailto:deyingkang@126.com).

Correspondence to: Department of Evidence-based Medicine and Clinical Epidemiology, the 8th teaching building, West China Hospital, Sichuan University, Chengdu, China ,610041. Tel./fax: +0086 28 85423691.

**Supplemental methods**

**Search strategy**

**PART ONE**: “pre-appraised” evidence resources

1. American College of Physicians Journal Club

(ACPJC, http://plus.mcmaster.ca/acpjc),

2. Evidence Update

(<http://plus.mcmaster.ca/evidenceupdates>),

3. Cochrane Library

([http://www.thecochranelibrary.com](http://www.thecochranelibrary.com/)),

4. Database of Abstracts of Reviews of Effects

(DARE, http://www.crd.york.ac.uk/crdweb/home.aspx?DB=DARE),

5. The Campbell Library (<http://www.campbellcollaboration.org/library.php>),

6. TRIP database

([http://www.tripdatabase.com](http://www.tripdatabase.com/)) and

7. Stroke

([http://stroke.ahajournals.org](http://stroke.ahajournals.org/)).

**PART TWO:**

The following search strategy,

(See: ‘Specialized register’ section in Cochrane Stroke Group: <http://onlinelibrary.wiley.com/o/cochrane/clabout/articles/STROKE/frame.html>)

using a combination of controlled vocabulary and text word terms, was used for MEDLINE and was modified to suit other databases.

MEDLINE (Ovid)

1 exp cerebrovascular disorders/

2 (stroke$ or poststroke$ or cva$).tw.

3 (cerebrovascular$ or cerebral vascular).tw.

4 (cerebral or cerebellar or brainstem or vertebrobasilar).tw.

5 (infarct$ or isch?emi$ or thrombo$ or apoplexy or emboli$).tw.

6 4 and 5

7 (cerebral or intracerebral or intracranial or parenchymal).tw.

8 (brain or intraventricular or brainstem or cerebellar).tw.

9 (infratentorial or supratentorial).tw.

10 7 or 8 or 9

11 (haemorrhage or hemorrhage or haematoma or hematoma).tw.

12 (bleeding or aneurysm).tw.

13 11 or 12

14 10 and 13

15 1 or 2 or 3 or 6 or 14

16 acupuncture/

17 exp acupuncture therapy/

18 electroacupuncture/

19 meridians/

20 acupuncture points/

21 acupuncture$.tw.

22 (electroacupuncture or electro- acupuncture).tw.

23 acupoints.tw.

24 ((meridian or non-meridian or trigger) adj10 point$).tw.

25 or/16-24

26 15 and 25

| Supplemental Table 1. The strength of recommendations | |
| --- | --- |
| Strength of recommendations | Comment |
| **Strong recommendation** |  |
| Strong recommendation for acupuncture | We are *confident* that the desirable effects of acupuncture outweigh its undesirable effects |
| Strong recommendation against acupuncture | We are *confident* that the undesirable effects of acupuncture outweigh its desirable effects |
| **Weak recommendation** |  |
| Weak recommendation for acupuncture | The desirable effects *probably* outweigh the undesirable effects |
| Weak recommendation against acupuncture | The undesirable effects *probably* outweigh the desirable effects |

| Supplemental Table II. Characteristics of included systematic reviews and their original RCTs | | | | | | | | |
| --- | --- | --- | --- | --- | --- | --- | --- | --- |
| **SRs** | **Original RCTs** | **Number of patients**(G1/G2/G3) | **Age**  (yrs) | **Gender** (M/F) | **Stroke type** | **Severity on entry** | **Time since stroke** | **Stage** |
| Wu 2006^46^ | Dai 1997^42^ | 136(46/45/45) | 48-86 | M:75% | Ischemic | L,Md,S | 3-14 mo | Recovery |
|  | Li 1997^43^ | 112(42/20/50) | 24-76 | na | Ischemic/hemorrhagic | na | 1mo-8.5yrs | Recovery |
|  | Lun 1999^44^ | 109(61/48) | 35-75 | M:60% | Ischemic/hemorrhagic | na | 2mo-5yrs | Recovery |
|  | Naeser 1992^55^ | 16(10/6) | 44-74 | na | Ischemic | Md | 1-3mo | Recovery |
|  | Wang 2001^60^ | 90(34/30/26) | 39-75 | M:56% | Ischemic/hemorrhagic | na | 2mo-5yrs | Recovery |
| Xie 2008^53^ | Han 2004^56^ | 66(34/32) | 41-79  (mean 62.1) | M:41;F:25 | ischemic/hemorrhagic | — | ＜30d | Subacute |
| Sze 2002^54^ | Hu 1993^12^ | 30(15/15) | T:Mean 63.6  (SD 6.7)  C:Mean 62.8  (SD 8) | T:15/0;  C:13/2 | ischemic/hemorrhagic | Md,S | ＜36h | Acute |
|  | Johansson 1993^57^ | 78(40/30) | Median:76 | na | Ischemic | Md,S | 4-7d | Acute |
|  | Sallstrom 1996^59^ | 45(24/21) | T:median 57  (35–69)  C:median 58  (39–72) | T:18/6;  C:16/5 | Ischemic/hemorrhagic | Md | median:40d (15-71d) | Subacute and recovery |
|  | Gosman-Hedstrom  1998^58^ | 104(37/34/33) | Weighted mean  (M 76;F:78.3) | 46/58 | Ischemic | Md,S | ＜7d | Acute |
|  | Wong 1998^60^ | 118(59/59) | 21-80 | T:38/21;  C:42/17 | Ischemic/hemorrhagic | Md,S | 10-14d | Acute |
|  | Sze 2002^61^ | 106(31/22/31/22) | mean 70.8  (SD 8.8) | 56/50 | Ischemic/hemorrhagic | Md,S | 3-15d | Acute |
|  | Tang 1996^62^ | 63(30/33) | 51-70 | 43/22 | Ischemic | na | ＜7d | Acute |
|  | Si 1998^63^ | 42(20/22) | T:mean 68  (SD 10)  C:mean 67  (SD 8) | T:15/5;  C:18/4 | Ischemic | Md | ＜7d | Acute |
|  | Jin 1999^64^ | 120(60/60) | 50-85 | 74/46 | Ischemic | na | ＜30d | Acute and subacute |
|  | Li 1999^65^ | 64(30/34) | 52-75 | T:17/13;  C:19/15 | hemorrhagic | Md | ≤2d | Acute |
|  | Zhang 1999^66^ | 241(145/96) | 35-85 | T:108/37;  C:71/25 | Ischemic/hemorrhagic | na | ＜43d | Subcute and recovery |
|  | Chou 2000^68^ | 32(16/16) | na | na | Ischemic/hemorrhagic | Md | 2d | Acute |
|  | Naeser 1992^55^ | 16(10/6) | 44-74 | na | Ischemic | Md | 1-3mo | Recovery |
|  | Johansson 2001^67^ | 150(48/51/51) | Weighted mean:76.3 | 14/136 | Ischemic | Md,S | 5-10d | Acute |
| Acute stage: stroke onset within 2 weeks of stroke onset;subacute stage:stroke onset within 2 to 28 days; recovery stage: stroke onset after 28 days. | | | | | | | | |
| Na, not available;L,light;Md,moderate;S,severe;F,female;M,male;mo,months;yrs,years;d,days;C,control group;T,treatment group;SD, standard deviation; SRs, systematic reviews;RCTs, randomized controlled trial; G1,Group1;G2,Group 2;G3,Group 3. | | | | | | | | |

| Supplemental Table II. Characteristics of included systematic reviews and their original RCTs (continued) | | | | | | |
| --- | --- | --- | --- | --- | --- | --- |
| **SRs** | **Original RCTs** | **Interventions** | | **Primary outcomes** | **Follow-up during** | **Conclusion** |
|  |  | **Treatment group** | **Control group** |  |  |  |
| Wu 2006^46^ | Dai 1997^42^ | Acupuncture+Aspirin25 mg qd;30d | Aspirin25 mg qd;30d | Neurological improvement^†^ | 30d | Positive |
|  | Li 1997^43^ | Acupuncture+PT and OT(6 times /wk,12wk) | PT and OT(6times/wk,12wk) | Neurological improvement^*^ | 3mo | Positive |
|  | Lun 1999^44^ | Acupuncture+TCM qd;45d | TCM qd;45d | Neurological improvement^†^ | 45d | Positive |
|  | Naeser 1992^55^ | Acupuncture(20 min/times,5 times/wk,1-2Hz)+ rehabilitation therapy,4wk | Sham acupuncture(20min/times,5times/wk,1-2 Hz)+ rehabilitation therapy,4wk | BMIT | 35d | Positive |
|  | Wang 2001^60^ | Acupuncture+TCM (10 times/13d,6 times) | TCM (10 times/13d,6 times) | Neurological improvement^†^ | 78d | Positive |
| Xie 2008^53^ | Han 2004^56^ | Acupuncture+basic treatments qd;63d | Basic treatments qd;63d | Swallowing function(CSRS) | — | Positive |
| Sze 2002^54^ | Hu 1993^12^ | Electrical acupuncture(30–60min/times,3times/wk,9.4 Hz),4wk | Basic treatments,4wk | SSS;  BI-100 | 7,14,21,28,90 d | SSS positive;  BI negative |
|  | Johansson 1993^57^ | Acupuncture(Classic acupuncture+Electrical acupuncture,30min/times,2times/wk, 2-5 Hz)+ PT and OT,10wk | PT and OT,10wk | Mobility Score;  BI-100 | Mobility Score:1, 3 mo;  BI:1,3,12mo | Motor negative;  BI positive |
|  | Sallstrom 1996^59^ | Acupuncture(Classic acupuncture,electrical acupuncture or moxibustion,30min/times,3-4 times/wk, 2-4Hz)+ rehabilitation therapy,6wk | Rehabilitation therapy,6wk | MAS;  SADLI | 6wk | MAS positive;  SADLI  positive |
|  | Gosman-Hedstrom  1998^58^ | Acupuncture(Classic acupuncture+electrical acupuncture,30min/times,2times/wk,2Hz)+PT and OT,10wk | PT and OT,10wk | SSS;  BI-100;  SADLI | 3wk,3mo,12mo | SSS negative;  BI negative |
|  | Wong 1998^60^ | Electrical acupuncture(30min/times,5times/wk, 20-25Hz, 10-20 mV)+PT and OT (≥2h/d),2wk | PT and OT (≥2h/d),2wk | BS;  FIM | T:mean 29.1(SD  7.9) d;  C: mean 32.4 (SD 8.2)d | BS positive; FIM positive |
|  | Sze 2002^61^ | Acupuncture [30min/times,3times/wk (outpatients) or 5 times/wk (inpatients)]+PT,OT,ST,10wk | PT,OT,ST,10wk | FMAM;  FIM;  BI-20 | 0,5,10wk | FMAM  Negative;  BI negative |
|  | Tang 1996^62^ | Acupuncture(200 times/min, acupuncture 5 mins and then 3-minute intervals,3times)+basic treatments,15d | Basic treatments,15d | Muscle power;  2nd NCDC disability score | 2,15d | 2^nd^ NCDC disability score positive |
|  | Si 1998^63^ | Acupuncture(classic acupuncture+electrical acupuncture,30min/times,5times/wk,5/45 Hz, 3.0mA)+basic treatments (heparin, low molecular dextran and nimodipine),36d | Basic treatments (heparin, low molecular dextran and nimodipine),36d | CSS | T: mean 37(SD 12)d;  C:mean 36(SD 13)d | positive |
|  | Jin 1999^64^ | Acupuncture(classic acupuncture +electrical acupuncture,50min/times,5times/wk, 5/45 Hz, 7.5mA)+basic treatments+TCM,40d | Basic treatments+TCM,40d | 2nd NCDC impairment score;  2nd NCDC disability score | 40d | 2^nd^ NCDC disability score positive |
|  | Li 1999^65^ | Acupuncture(30min/times,qd)+basic treatments | Basic treatments | 2nd NCDC impairment score;  2nd NCDC disability score | na | 2^nd^ NCDC disability score positive |
|  | Zhang 1999^66^ | Acupuncture(40min/times,qd)×20-30times+ basic treatments | Basic treatments | UNCHMS Guideline | na | UNCHMS positive |
|  | Chou 2000^68^ | Acupuncture(acupuncture 3 mins and then 5-minute inter-vals,2 times)+basic treatments,20d | Basic treatments,20d | MESSS | na | MESSS positive |
|  | Naeser 1992^55^ | Acupuncture(20 min/times,5 times/wk,1-2Hz)+ rehabilitation therapy,4wk | Sham acupuncture(20min/times,5times/wk,1-2 Hz)+ rehabilitation therapy,4wk | BMIT | 35d | BMIT negative |
|  | Johansson 2001^67^ | Acupuncture+ PT,OT,ST,10wk | G1: transcutaneous electrical nerve stimulation+PT,OT,ST,10wk,  G2: transcutaneous electrical nerve stimulation (Low intensity and high frequency:80 Hz, 0.4 mA)+PT,OT,ST,10wk | RMI;  BI-100 | 0,3,12mo | RMI negative;  BI-100 negative |
| * CSRS 1 and † CSRS 2(the two versions of Chinese Stroke Recovery Scale, based on the revised diagnostic criteria of acute cerebral infarction formulated by the second or based on principles of traditional Chinese medicine)  G1,Group1;G2,Group 2;G3,Group 3;na, not available; qd, once a day; d,day;PT, physical therapy; OT, occupational therapy; wk, week; mo, months; BMIT, Boston Motor Inventory Test; SSS, Scandinavian Stroke Scale; BI, Barthel Index; MAS, Motor Assessment Scale; SADLI, Sunnaas ADL Index; h, hours; BS, Brunnstrom Stages; FIM, Functional Independence Measure; SD, standard deviation; ST, speech therapy; FMAM, Fugl-Meyer Assessment Motor score; NCDC, National Cerebrovascular Diseases Conference; CSS, Chinese Stroke Scale; UNCHMS, Use of New Chinese Herbal Medicines in Stroke; MESSS, Modified Edinburgh-Scandinavian Stroke Scale; RMI, Rivermead Mobility Index; min, minute; TCM, traditional Chinese medicine; SRs, systematic reviews; RCTs, randomized controlled trial; CSRS, Chinese Stroke Recovery Scale. | | | | | | |
